# Effective Cataract Surgery Coverage and Potential Influencing Factors for Adults in Fujian, Southeast of China: Fujian Eye Study

**DOI:** 10.1155/bmri/6690380

**Published:** 2026-04-08

**Authors:** Qinrui Hu, Xiangdong Luo, Dasen Xie, Shengqi Su, Bin Wang, Zhaode Zhang, Yang Li, Xiaoxin Li

**Affiliations:** ^1^ Xiamen Eye Center and Institute of Xiamen University, Xiamen, China; ^2^ Xiamen Clinical Research Center for Eye Diseases, Xiamen, Fujian, China; ^3^ Xiamen Key Laboratory of Ophthalmology, Xiamen, China; ^4^ Fujian Key Laboratory of Corneal & Ocular Surface Diseases, Xiamen, Fujian, China; ^5^ Xiamen University Affiliated Keji High School, Xiamen, Fujian, China; ^6^ Ningde Normal University Affiliated Ningde Hospital, Ningde, Fujian, China; ^7^ Department of Ophthalmology, Peking University People′s Hospital, Beijing, China, pku.edu.cn; ^8^ Xiamen Key Laboratory of Corneal & Ocular Surface Diseases, Xiamen, Fujian, China; ^9^ Translational Medicine Institute of Xiamen Eye Center of Xiamen University, Xiamen, Fujian, China

**Keywords:** adults, china, effective cataract surgery coverage, epidemiology, influence factors

## Abstract

**Purposes:**

The purpose of this study is to identify the impact factors of effective cataract surgery coverage (eCSC) through a cross‐sectional epidemiological survey for the population in the southeast of China.

**Methods:**

The eCSC for primary analysis was defined as the proportion of the population that underwent cataract surgery and achieved a good visual outcome (6/12 or better) in the Fujian Eye Study (FJES). The FJES is a cross‐sectional epidemiological survey targeting individuals aged 50 years and older in southeastern China. Multivariate analysis was performed to identify potential factors associated with eCSC. The study primarily examined socioeconomic status, geographical location, and various sociodemographic characteristics within this region.

**Results:**

In this study, the eCSC rates were 34.7%, 51.0%, and 92.6% based on visual acuity thresholds of 6/12, 6/18, and 6/60, respectively. Univariate analysis revealed that males had significantly higher eCSC rates compared with females (38.5% vs. 31.9%, *p* = 0.046). Higher educational attainment was associated with increased eCSC rates (*p* = 0.005). Similarly, residents with higher incomes demonstrated significantly higher eCSC rates (51.6%) compared with those with lower incomes (29.6%, *p* = 0.046). Additionally, individuals with a history of alcohol consumption showed a higher prevalence of cataract surgery (51.6% vs. 29.6%, *p* = 0.005). Multivariate linear regression analysis identified age and educational level as significant predictors of eCSC.

**Conclusion:**

Age and educational level emerged as critical factors associated with eCSC. These findings highlight the influence of sociodemographic variables on cataract surgery outcomes in the population.

## 1. Introduction

Cataracts remain the predominant global cause of blindness, with surgical intervention as the primary modality of treatment. Elevating the volume of cataract surgeries and evaluating their efficacy in alleviating blindness have been prioritized as critical objectives on the global health agenda [1]. The cataract surgical coverage (CSC) metric, which has been integral to ophthalmic health surveys for over two decades, quantifies the proportion of individuals within a population who have undergone or are in need of cataract surgery [2]. The effective cataract surgery coverage (eCSC), introduced in 2017, represents a refined metric that accounts for the quality of surgical outcomes by incorporating postoperative visual acuity [2]. The eCSC has since been recognized as a more sophisticated indicator. Population‐based eCSC metrics are vital for assessing the global provision and quality of eye care services [3], as well as for tracking advancements toward the goal of universal health coverage (UHC) [4]. Importantly, these indicators provide insights into not only the reach of cataract surgery but also the accessibility and standard of care, thereby ensuring that patients receive timely and effective treatment [5].

However, there is still limited evidence on the factors influencing eCSC, as highlighted in a recent review [6]. The data used in the review were sourced from only two high‐income countries, which limits their global applicability. The review also emphasized the need to address gender disparities in eCSC across regions, in addition to focusing on other underserved groups, in order to achieve more equitable improvements. To effectively track eCSC progress, it is essential to update the evaluation methods. Since high‐income countries tend to have higher eCSC rates, regional findings may underestimate the true levels. Therefore, more population‐based surveys are required, with an emphasis on expanding geographic coverage to fill existing data gaps through 2030.

In this study, we examined the baseline and key factors influencing population eCSC, updated indicators of regional economic and biological correlations, and refined the regional eCSC data.

## 2. Methods

Cataracts among residents were investigated in relation to socioeconomic status, geographical location, and other sociodemographic factors as part of the Fujian Eye Study (FJES), a cross‐sectional epidemiological survey conducted from May 2018 to October 2019. This study involved 8211 adults aged 50 years and older from both urban and rural areas of southern China [7]. In this study, a stratified, multistage, cluster random sampling method was applied in southern China. Communities were randomly selected from both urban and rural strata. Within each selected cluster, residents aged ≥ 50 years were invited to participate. The response rate was 81.8% (8211/10,044).

eCSC was estimated based on factors above. In this analysis, eCSC was defined as the proportion of the population who underwent cataract surgery with a good outcome (6/12 for visual acuity). And a preliminary evaluation with other assessment criteria (6/18 and 6/60) was also conducted in the study.

The eCSC formula was as follows:

eCSC = (*a* + *b*)/(*x* + *y* + *z*),where “*a*” refers to individuals with unilateral operated cataracts who attained a specified threshold of postoperative visual acuity in the operated eye and had vision impairment (using BCVA∗) in the other eye; and “*b*” represents individuals with bilateral operated cataracts who attained a specified threshold of postoperative visual acuity in at least one eye. “*x*” indicates individuals with unilateral pseudo/aphakia and operable cataracts in the other eye; “*y*” indicates individuals with bilateral pseudo/aphakia regardless of visual acuity; and “*z*” indicates individuals with bilateral operable cataracts.

## 3. Statistical Analysis

Statistical analysis in this study was performed with Stata software (Version 15.1, Stata Corp. LLC, College Station, Texas, United States). Residents were grouped by sex, age, education level, income, urban or rural residence, and coastal and inland residence. Comparisons of different groups were performed by analysis of variance (ANOVA). A multifactorial analysis was performed for the numbers of “*a*” and “*b*” individuals in the overall cataract population. Two‐tailed *p* values less than 0.05 were considered to indicate statistical significance.

## 4. Results

### 4.1. Population

A total of 8211 residents (response rate: 81.8%; 8211 out of 10,044) aged 50 years and older were included in the study. Of these, 4836 (58.9%) were female, and 4678 (57.0%) were from urban areas. The mean age was 64.39 years (SD = 8.87; median = 64; range: 50–98 years).

Among the participants, 652 individuals had undergone cataract surgery, including 272 with bilateral surgery and 380 with unilateral surgery, accounting for 7.94% of the population (*a*: 56, *b*: 178, *x*: 138, *y*: 272, and *z*: 264). The mean age of these individuals was 73.32 years (SD = 8.79), with a range of 50–98 years. Statistical analysis was performed to assess the eCSC.

### 4.2. eCSC and Visual Outcomes

**Figure 1:**
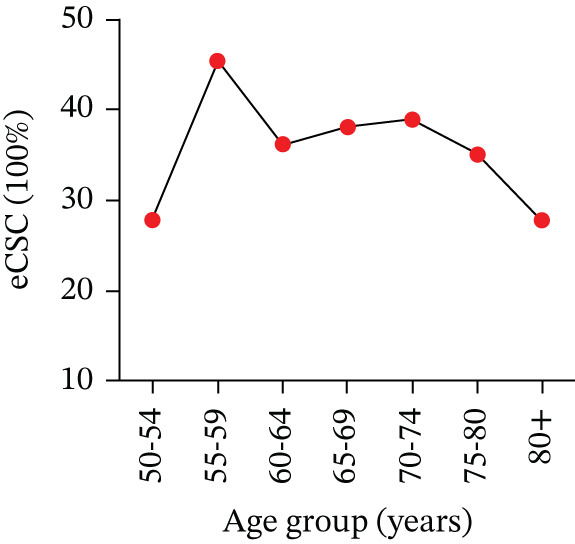
ECSC by age group.

In this study, the threshold for eCSC was defined as 6/12 visual acuity, with 34.72% of individuals achieving good outcomes. eCSC was significantly higher in males (38.5%) compared with females (31.9%, *p* = 0.043). Table 1 provides a detailed summary of participant characteristics, including total sample size, sex, region, age group, height, weight, body mass index (BMI), heart rate (HR), systolic blood pressure (SBP), diastolic blood pressure (DBP), education level, and income group. There was notable variation in eCSC by income, as illustrated in Figure 1. As income levels increased, eCSC also rose significantly (*p* = 0.046).

**Table 1:** Baseline characteristics of the study population.

Characteristics	Total (*x* + *y* + *z*)	*a* + *b*	(*x* + *y* + *z*)−(*a* + *b*)	*p*
	(*n* = 674)	(*n* = 234)	(*n* = 440)	
Demographics				
Age (years)	73.32 ± 8.79	72.25 ± 7.98	73.90 ± 9.15	0.020
Sex				
Female, *n* (%)	386 (57.3%)	123 (31.9%)	263 (68.1%)	
Male, *n* (%)	288 (42.7%)	111 (38.5%)	177 (61.5%)	0.043
Medical history				
HBP	475 (70.5%)	157 (33.1%)	318 (66.9%)	0.183
Smoking, *n* (%)	98 (18.2%)	40 (40.8%)	58 (59.2%)	0.130
Alcohol consumption, *n* (%)	63 (12.1%)	32 (50.8%)	31 (49.2%)	0.005
Diabetes, *n* (%)	188 (27.9%)	68 (36.2%)	120 (63.8%)	0.343
Hyperlipidemia, *n* (%)	97 (14.4%)	38 (39.2%)	59 (60.8%)	0.189
Social factors				
Education level				
Primary school and below	202 (35.5%)	50 (24.8%)	152 (75.2%)	
Junior high school	140 (24.6%)	51 (36.4%)	89 (63.6%)	
Senior high school	176 (30.9%)	65 (36.9%)	111 (63.1%)	
College and above	51 (9.0%)	29 (56.9%)	22 (43.1%)	0.005
Income (yuan, RMB)				
< 2000	240 (62.8%)	71 (29.6%)	169 (70.4%)	
2000–5000	111 (29.1%)	35(31.5%)	76 (68.5%)	
> 5000	31 (8.1%)	16 (51.6%)	15 (48.4%)	0.046
Urbanization				
Urban	329 (48.8%)	115 (35.0%)	214 (65.0%)	
Rural	345 (51.2%)	119 (34.5%)	226 (65.5%)	0.482

**Figure 2:**
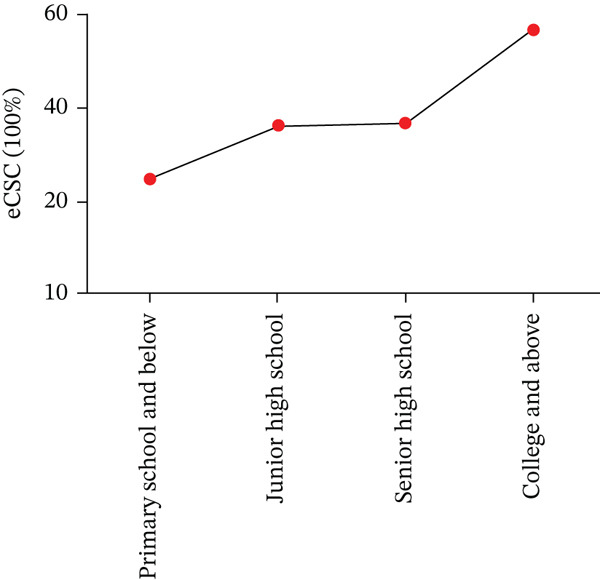
ECSC by age education level.

**Figure 3:**
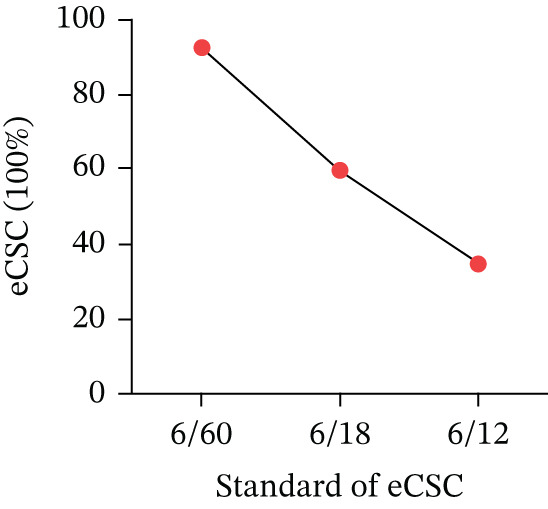
ECSC by different standards in vision acuity.

Regarding education, eCSC varied by educational attainment (Figure 2). The eCSC was 24.8% among the illiterate group and 56.9% among those with a college education or higher (*p* = 0.005). No significant difference in eCSC was observed by smoking status (*p* = 0.243) or blood pressure (*p* = 0.183). However, alcohol consumption was associated with a difference in eCSC (*p* = 0.005). At different visual acuity thresholds, eCSC values were 34.7% (6/12), 51.0% (6/18), and 92.6% (6/60), respectively (Figure 3).

### 4.3. Predictors of eCSC

In multivariate analysis, higher education level was the only significant predictor of increased eCSC (Exp[B] = 1.72, 95% CI 1.25–2.37; *p* = 0.001). Age was negatively associated with eCSC (Exp[B] = 0.97, 95% CI 0.94–1.00; *p* = 0.039). Other variables—including gender, diabetes, hypertension, hyperlipidemia, smoking, alcohol use, income, and residence—were not significantly related to eCSC (Table 2).

**Table 2:** Predictors of increasing eCSC by multivariate logistic regression.

	B	*p*value		OR (Exp[B])	95.0% CI	
					Lower	Upper
Gender	0.040	0.990	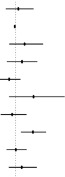	1.004	0.560	1.800
Age	−0.032	0.039		0.968	0.939	0.998
Diabetes	0.235	0.417		1.265	0.717	2.231
Hypertension	0.157	0.572		1.170	0.679	2.017
Hyperlipidemia	−0.492	0.163		0.612	0.307	1.219
Alcohol consumption	0.409	0.285		1.506	0.711	3.192
Smoking	−0.343	0.366		0.709	0.337	1.494
Education level	0.541	0.001		1.718	1.246	2.368
Income	−0.060	0.800		0.942	0.591	1.500
Urban	0.157	0.548		1.170	0.701	1.951
Constant	0.282	0.822		1.326		

*Note:* B represents the log odds coefficient from the multivariable logistic regression model.

Abbreviations: CI, confidence interval; OR, odds ratio.

## 5. Discussion

Age and education were strongly associated with eCSC in our study, which systematically assessed the impact of biological, socioeconomic, geographic, and lifestyle factors on cataract surgery coverage. In contrast, other factors such as income, rural–urban differences, systemic disease status, and sex were not significantly linked to eCSC rates. A discrepancy was observed between the univariate and multivariate analyses. This difference can be explained by confounding and collinearity among sociodemographic variables. In our population, age was closely correlated with both education and income; therefore, its independent effect on eCSC was masked in the univariate analysis. After adjusting for these interrelated factors, the effect of age became evident, whereas the apparent associations of sex, income, and alcohol use were largely explained by differences in age and education. These findings indicate that age and educational attainment are the primary determinants of eCSC, whereas other demographic and lifestyle factors may exert only indirect or secondary influences through socioeconomic pathways.

The overall eCSC in this study was 34.7%, using the 6/12 visual acuity threshold, which is comparable with the rate in Europe (37.7%) but slightly lower than that in Southeast Asia (40.4%) [6]. A preliminary analysis (unpublished) of 47 population‐based surveys from 11 countries revealed a wide variation in eCSC, ranging from 2.8% to 88.5%. In a study covering 55 countries, the median eCSC was 24.8%, with considerable regional variability. The eCSC rate observed in our study was moderate, reflecting regional trends [1, 6].

In this study, eCSC was inversely related to the threshold for a good outcome after cataract surgery. The eCSC for the 6/18 threshold (51.0%) was lower than for the 6/60 threshold (92.6%), and the overall effective surgery rate for the 6/12 threshold (34%) was significantly lower than for the higher thresholds. These findings align with results from multiple studies. Given the need for visual quality improvement and the prevention of vision loss, the WHO has recommended that countries report effective coverage using the cataract surgery threshold most appropriate to their context [8]. Our results underscore that there are still regions where individuals with cataract‐induced blindness lack access to services. Monitoring eCSC at multiple cataract surgical thresholds, as shown in our study, is critical. We also found that additional efforts are needed to improve outcomes in regions with the 6/12 threshold.

Notably, in our study, eCSC was lower in the 50–55 and over 80 age groups, with the highest rates observed in middle‐aged individuals, whereas few published studies have examined eCSC stratified by age. A similar study found that younger patients (aged < 75 years) were more likely to undergo cataract surgery than older patients, which aligns with findings from previous studies on willingness to pay (WTP) for cataract surgery in this region [9]. Cataract surgery was significantly more common in younger populations, likely due to higher surgical expectations and socioeconomic status [10]. Interestingly, our study also revealed that younger patients (around 50 years old) had lower cataract surgery rates. This may be due to early detection of cataracts in this age group, limited access to medical support, lack of awareness about the surgery, and misconceptions about its outcomes. Additionally, social medical screenings have generally been more prevalent among older populations, with many screenings occurring only after retirement, as confirmed by multiple studies [11].

Additionally, individuals aged ≥ 75 years, who are often not employed and lack a steady income, are more likely to place less value on improved vision. Therefore, it is important to prioritize improving eCSC, especially for individuals at both ends of the age spectrum. For the elderly, comorbidities, financial constraints, and lower perceived benefit may contribute to the above results. Targeted interventions such as early screening, health education, and enhanced insurance support are warranted.

Our study found that eCSC was higher among individuals with formal education. Specifically, the university‐educated population had a higher eCSC, whereas the illiterate population had a lower eCSC. This finding aligns with results from the Tehran Geriatric Eye Study, which showed that cataract surgery rates were significantly inversely related to education level (OR = 0.55, *p* = 0.006 for college education vs. illiteracy) [12]. Patients with better health knowledge were more willing to pay for cataract surgery, and they were willing to pay more. These findings are consistent with a study conducted in China [11]. It suggests that educated individuals may place a higher value on their vision and have a better understanding of the cost‐benefit aspects of healthcare services [13].

Growing evidence underscores the importance of awareness and knowledge of cataracts in increasing demand for surgery, particularly in rural China [9]. In a community‐based cross‐sectional study, 379 participants (64.7%) demonstrated good knowledge of cataracts. Factors positively associated with cataract knowledge included age (≥ 40 years), completion of elementary school, high school education or higher, government or nongovernment employment, merchant occupation and a positive attitude. However, rural residence was negatively associated with cataract knowledge [14]. Our study highlights that adequate educational awareness plays a crucial role in improving eCSC. Individuals with formal education are not only more proactive in seeking healthcare but are also better equipped to utilize medical resources, leading to higher surgery rates. Therefore, integrating education as a key intervention is vital for improving both the quality and accessibility of cataract services [15].

Despite variations in eCSC across different income groups, this study found little impact from rural–urban differences and income levels on eCSC. In China, cataract surgery is widely accessible and affordable at medical institutions across all levels, with government support available to patients. This could explain why income did not significantly influence eCSC between urban and rural areas. However, in a study by Hashemi et al., cataract surgery was significantly associated with economic status and age (OR: 14.06; *p* < 0.001 for > 80 vs. 60–64 years) [12]. Similarly, a study on WTP found that both individual and household characteristics contributed to a lower WTP for cataract surgery in similar areas [16]. Evidence on interventions to improve cataract services highlights regional inequalities, underscoring the need for more data from low‐ and middle‐income countries that address all quality aspects, including planetary health [17]. Income adequacy reflects a socioeconomic gradient in CSC or eCSC at various cataract surgery thresholds [18].

There was a significant disparity in cataract surgery prevalence between wealthier and poorer patients. Age, economic status, and education had the largest effect on increasing inequality, whereas a history of eye exams and insurance coverage were key factors in reducing this disparity [12, 19–21]. Wide variation in eCSC by country and a gradient of increasing eCSC with higher World Bank income levels reflect the tendency for wealthier nations to have more resources, leading to greater cataract service output [6].

There was no statistically significant association between sex and eCSC in our study. These findings align with previous studies in Latin America, where no significant sex‐related difference in eCSC was found. Similarly, there was no evidence of a pooled difference in male and female eCSC or CSC in the Americas or Europe [6]. However, some studies have concluded the opposite, showing that men were more likely to undergo cataract surgery than women, particularly in low‐ and middle‐income countries such as South Asia and India [22–24]. These studies confirm that women tend to have lower coverage, and that gender inequities in eCSC remain an issue. Higher levels of inequality were observed in eCSC compared with CSC, highlighting the compounded disadvantage women face in terms of both access to surgery and postsurgical visual outcomes [2]. Thus, addressing gender disparities in eCSC is critical, especially in low‐ and middle‐income countries, where underserved groups must also be prioritized to improve equity in eye care [25].

There was minimal association between an individual′s systemic disease status (hypertension and diabetes) or unhealthy habits and eCSC in our study. Although systemic conditions can increase the incidence of cataracts and negatively impact postsurgical visual acuity [26, 27], they did not significantly influence eCSC rates. Therefore, although systemic disease may increase cataract incidence, it is not a major factor affecting the rate of effective cataract surgery.

Our findings are generally consistent with those reported from other middle‐income regions, including India, Latin America, and parts of Africa, where eCSC remains suboptimal despite increasing surgical capacity [6]. Similar socioeconomic gradients—by age, education, and residence—have been observed across these populations, suggesting that barriers to achieving optimal eCSC are largely systemic rather than clinical. In China, several large‐scale national initiatives, such as the National Program for the Prevention and Treatment of Blindness and the Cataract Surgery Subsidy Scheme, have improved affordability and accessibility of cataract services, especially in rural areas [26]. Together with the expansion of universal medical insurance, these policies have narrowed economic gaps in surgical access, yet regional and quality‐of‐care disparities persist. Our results reinforce the need to shift the national focus from surgical quantity to surgical effectiveness—fully aligning with the World Health Organization′s goal of improving eCSC as a core indicator of progress toward UHC [15].

Several limitations exist in our study. Only the 6/12 threshold for operable cataracts and good outcomes was used; other thresholds were not fully explored, and the potential influencing factors may vary across different baselines. As recommended by the WHO, eCSC rather than CSC was used as the primary indicator in this study. Our research systematically examined eCSC among residents, providing valuable insights for improving eCSC rates. However, the study was insufficient to offer a comprehensive overview and comparison of global and regional eCSC characteristics. Additional research is needed to explore differences between various thresholds and regions. As a cross‐sectional design, the study can demonstrate associations rather than establish causal relationships between sociodemographic factors and eCSC. Longitudinal or interventional studies are needed to confirm the causal pathways underlying these associations.

In summary, this population‐based study revealed that eCSC in southeastern China remains suboptimal. Among various sociodemographic and lifestyle factors, age and educational attainment were identified as the primary determinants of eCSC, whereas other factors showed limited independent influence. Understanding these determinants is essential for developing targeted interventions to improve both the accessibility and quality of cataract services. Strengthening health education, expanding early screening, and prioritizing underserved populations can enhance eCSC and contribute to achieving UHC goals. And the influencing factors of eCSC warrant further investigation through longitudinal and interventional studies.

## Author Contributions

Qinrui Hu and Xiangdong Luo made equal contributions to all aspects of the study, including the study design, data analysis, preparation of related data, and writing and revision. Dasen Xie and Shengqi Su were mainly engaged in data collation. Bin Wang assisted in article revision and plotting. Xiaoxin Li and Yang Li oversaw the research design, data revision, and manuscript writing. Thanks to Springer Nature for their professional language service. We thank all FJES group members (Zhenglingling Yao, Liting Wang, Yi Liu, Wufu Qiu, Menging Lin, and Yanhong Zhang) who made tremendous efforts to make the study successful, especially in the field examinations and data collection.

## Funding

This study was supported by National Natural Science Foundation of China (10.13039/501100001809) (81870672) and Natural Science Foundation of Xiamen Municipality (10.13039/100016808) (3502Z20227290).

## Ethics Statement

Our study was conducted in accordance with the Declaration of Helsinki. The study was approved by the Ethics Committee of Eye Institute and Affiliated Xiamen Eye Center of Xiamen University (No. XMYKZX‐KY‐2018‐001). All patients gave their informed consent to participate in the study.

## Consent

Consent was obtained directly from patients.

## Conflicts of Interest

The authors declare no conflicts of interest.

## Data Availability

The data that support the findings of this study are available on request from the corresponding authors. The data are not publicly available due to privacy or ethical restrictions.
